# Robotic-Assisted Laparoscopic “Salvage” Rectopexy for Recurrent Ileoanal J-Pouch Prolapse

**DOI:** 10.1155/2010/790462

**Published:** 2010-04-18

**Authors:** Madhu Ragupathi, Chirag B. Patel, Diego I. Ramos-Valadez, Eric M. Haas

**Affiliations:** Division of Minimally Invasive Colon and Rectal Surgery, Department of Surgery, University of Texas Medical School at Houston, 7900 Fannin Street, Suite 2700, Houston, TX 77054, USA

## Abstract

Total restorative proctocolectomy with ileal pouch-anal anastomosis (RP/IPAA) has become the standard of care for the surgical treatment of ulcerative colitis. Despite its correlation with an excellent quality of life and favorable long-term outcomes, RP/IPAA has been associated with several complications. Prolapse of the ileoanal pouch is a rare and debilitating complication that should be considered in the differential diagnosis of pouch failure. Limited data exist regarding the prevalence and treatment of pouch prolapse. We present the case of a recurrent J-pouch prolapse treated with a novel minimally invasive “salvage” approach involving a robotic-assisted laparoscopic rectopexy with mesh.

## 1. Introduction

Surgical intervention is required in 30%–40% of patients with chronic ulcerative colitis (UC) [1]. Total (restorative) proctocolectomy with ileal pouch-anal anastomosis (RP/IPAA) has become the standard of care for the surgical treatment of intractable UC [2]. Restorative proctocolectoy with IPAA restores bowel continuity with preservation of anorectal continence [3, 4], and is associated with improved quality of life and favorable long-term outcomes [5, 6]. Even so, this procedure has been associated with significant complications of the pouch itself, including pouchitis, stricture, obstruction, and pouch failure [7–9]. Many of these complications require surgical correction, the approaches of which have been well described [10]. However, prolapse of the ileoanal pouch is a rare and debilitating phenomenon following RP/IPAA. Limited experience and data exist with respect to the prevalence and surgical repair of pouch prolapse. We present the case of a recurrent J-pouch prolapse treated with an innovative robotic-assisted laparoscopic “salvage” procedure.

## 2. Materials and Methods

### 2.1. Case History

A 24-year-old female presented with an 8-month history of recurrent prolapse of an ileoanal J-pouch in May 2009. Her past medical history was significant for UC diagnosed at age 15. She became recalcitrant to medical therapy and underwent RP/IPAA and diverting ileostomy in 2005. The patient encountered no immediate complications following ileostomy reversal; however, within a year she experienced a sensation of bulging following each bowel movement and was diagnosed with a full-thickness prolapse of her J-pouch. She underwent surgical correction with a trans-abdominal suture repair of the prolapsing segment in 2007. The patient progressed well for almost one year until she presented to our institution with a recurrent pouch prolapse. 

**Figure 1:**
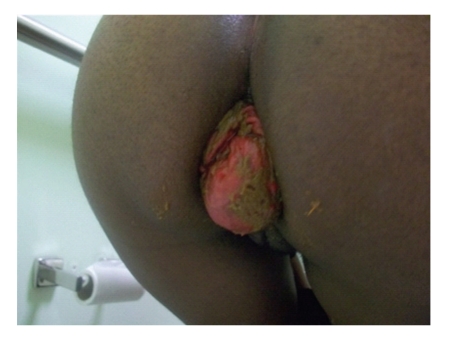
Prolapse of ileoanal J-pouch with straining, at initial visit.

The patient reported having 8–10 bowel movements per day and a full-thickness prolapse requiring manual reduction following each bowel movement. These episodes were associated with fecal incontinence and occasionally occurred with micturition. Anorectal examination revealed a patulous anus with diminished sphincter tone. When asked to strain, a full-thickness prolapse encompassing the entire pouch was readily visualized (see Figure 1). Endoscopy of the pouch demonstrated edematous mucosa without evidence of pouchitis. The patient underwent pelvic floor evaluation with anorectal physiologic studies, which indicated internal and external anal sphincter dysfunction with diminished mean manometric pressures of 35–40 mm Hg (normal range: 40–80 mm Hg) and 45–65 mm Hg (normal range: 80–160 mm Hg), respectively. 

The patient was first advised to complete a course of pelvic muscle rehabilitation (PMR, modified biofeedback) to optimize her pelvic floor weakness. She was then scheduled for repair using a novel minimally invasive approach involving a robotic-assisted laparoscopic (RALS) rectopexy with mesh. The procedure and possible complications were described to the patient and informed consent was obtained.

### 2.2. Operative Procedure

**Figure 2:**
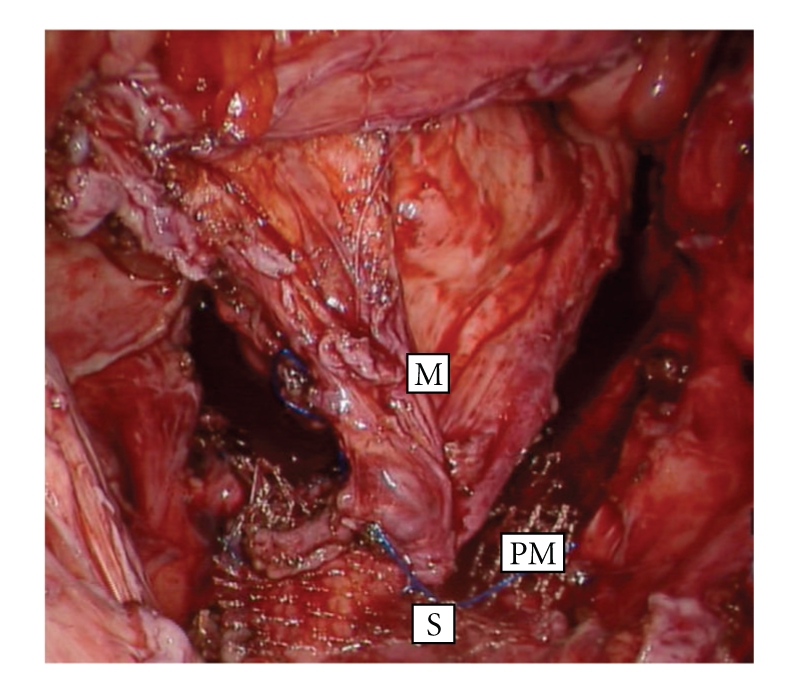
Suture rectopexy with mesh securing the mesentery of the pouch to the presacral fascia in the deep pelvis. The mesentery of the pouch (M), sacral promontory (S), and non-absorbable prolene mesh (PM) are labeled.

The procedure was performed by a board-certified colon and rectal surgeon (E.M.H.) using the da Vinci S-Type Surgical System (Intuitive Surgical, Inc., Sunnyvale, CA). The patient was placed in a modified lithotomy position with 10 degrees of flexion at the hips. A total of five trocars were utilized—a 12-mm camera port, three 8-mm robotic ports and a 5-mm accessory port for the assistant. Laparoscopic exploration revealed multiple abdominal and pelvic adhesions as well as a benign cystic mass in the pelvis. Multiple intraloop small bowel adhesions required careful adhesiolysis to gain access to the pelvis. Extensive ovariolysis was required as both fallopian tubes and ovaries were densely adhered to the prolapsing segment of the pouch. In addition a deep and redundant enterocele was encountered. Ultimately, the presacral plane was entered and the mesentery of the J-pouch was dissected to the level of the levator ani. It was noted during dissection that the mesentery of the pouch was torsioned 180 degrees and adhered to the sac of the enterocele in the anterior plane. Once the pouch mesentery was completely mobilized and returned to anatomical position, a suture rectopexy fastening the mesentery to the presacral fascia was performed. A non-absorbable prolene mesh was used to reinforce the rectopexy (see Figure 2). Care was taken to preserve the autonomic nerves of the pelvis as well as the vasculature of the pouch mesentery.

## 3. Results and Discussion

### 3.1. Results

The patient underwent robotic-assisted laparoscopic rectopexy without complication and did not require conversion to an open procedure. The robotic operative time was 128 minutes and the estimated blood loss was 200 mL. The patient had an expeditious recovery with return of bowel function, as evidenced by flatus, on postoperative day 2. At that point her diet was advanced, she was changed from intravenous to oral pain medication, and she was able to void following removal of her Foley catheter. She was discharged home on postoperative day 3. 

The patient presented to the office for postoperative evaluation at two weeks, six weeks, and three months following surgery. On two-week follow-up she continued to do well with no signs of complications. Repeat anal physiologic studies were performed six weeks following the procedure, and mean manometric pressures of the internal and external anal sphincters had improved to 45–65 mm Hg and 70–80 mm Hg, respectively. At three-month follow-up, she reported an overall excellent response with no further episodes of fecal incontinence or pouch prolapse.

### 3.2. Discussion

Restorative proctocolectomy with ileal pouch-anal anastomosis is an important treatment modality for patients suffering from refractory UC. Ileoanal pouch prolapse is a rarely reported complication following RP/IPAA. A review of the literature identified a total of 10 published reports of pouch prolapse in the adult population. Extent of prolapse varied from external mucosal or full-thickness prolapse [11–17] to internal intussusception [18, 19]. Some of the treatment modalities reported include fixation of the pouch to the presacral fascia and revision or excision with reconstruction. One report introduced the external pelvic neorectal suspension procedure in which transperineal placement of Permacol mesh was used to suspend the prolapsing segment [20]. 

Ehsan et al. estimated the prevalence of ileoanal pouch prolapse through a 2001 survey of the North American membership of the American Society of Colon and Rectal Surgeons [21]. Of 23, 541 procedures reported, a total of 83 patients (0.4%) presented with prolapse-related symptoms (e.g., external prolapse of tissue, straining, seepage, incontinence, and pain). Nearly half (48.2%) of these patients presented within 2 years of pouch construction, with a slight trend toward reduced incidence as more time had elapsed after surgery. Fifty-two of the patients (62.7%) underwent surgical correction through transanal, transabdominal or combined approaches; six of these (11%) involved mesh repair. Pouch salvage was achieved in 49 cases (94%), with pouch reconstruction being necessary in two cases and conversion to ileostomy in one case. Ehsan et al. identified several principal patterns and symptoms of presentation, and demonstrated the importance of considering pouch prolapse when evaluating potential causes of failure.

We report a novel robotic-assisted surgical approach in the treatment of a patient who presented with a J-pouch prolapse that had failed a previous suture rectopexy repair at another institution. Robotic surgery is an innovative technique requiring a high degree of technical training to overcome several of the challenges of this approach, most notably the loss of tactile and tensile feedback. Furthermore, a learning curve is required to develop understanding of the spatial relationships of the external robotic arms to avoid clashing of the instruments and inadvertent injury to the tissues. We chose a robotic approach as this technology facilitates the freedom of movement of an open surgical procedure while maintaining a minimally invasive platform. 

This surgical system provides a three-dimensional field of view, 10-fold magnification, and camera stabilization [22, 23], which optimized visualization in this re-operative field. In addition, the advanced robotic instrumentation with seven degrees of freedom of motion, tremor elimination and motion scaling [24] facilitated precise dissection in the previously operated presacral plane. Despite encountering dense small bowel adhesions, a large redundant enterocele, and torsioned pouch mesentery, we found this surgical approach most useful in maintaining the proper planes of dissection while preserving the critical structures of the pelvis (i.e. internal reproductive organs, ureters and vasculature). In light of the recurrent nature of the prolapse, we chose to reinforce the suture rectopexy with a non-absorbable prolene mesh.

## 4. Conclusion

We describe the case of a recurrent prolapsed J-pouch treated through a novel RALS “salvage” procedure. Due to the numerous benefits offered by this innovative system, we have been performing robotic-assisted surgery specifically for various colorectal procedures in which pelvic dissection is necessary. With current levels of experience limited to familiarity from case reports, ileal pouch prolapse may be overlooked in the differential diagnosis of pouch dysfunction. Robotic-assisted laparoscopic rectopexy with mesh proved to be feasible and safe for the correction of J-pouch prolapse.

## References

[1] Larson DW, Pemberton JH (2004). Current concepts and controversies in surgery for IBD. *Gastroenterology*.

[2] McGuire BB, Brannigan AE, O’Connell PR (2007). Ileal pouch-anal anastomosis. *British Journal of Surgery*.

[3] Mathur P, Hallan RI (2002). The colonic J-pouch in colo-anal anastomosis. *Colorectal Disease*.

[4] Bach SP, Mortensen NJM (2006). Revolution and evolution: 30 years of ileoanal pouch surgery. *Inflammatory Bowel Diseases*.

[5] Fazio VW, Ziv Y, Church JM (1995). Ileal pouch-anal anastomoses complications and function in 1005 patients. *Annals of Surgery*.

[6] Muir AJ, Edwards LJ, Sanders LL (2001). A prospective evaluation of health-related quality of life after ileal pouch anal anastomosis for ulcerative colitis. *American Journal of Gastroenterology*.

[7] Fazio VW, O’Riordain MG, Lavery IC (1999). Long-term functional outcome and quality of life after stapled restorative proctocolectomy. *Annals of Surgery*.

[8] Belliveau P, Trudel J, Vasilevsky C-A, Stein B, Gordon PH (1999). Ileoanal anastomosis with reservoirs: complications and long-term results. *Canadian Journal of Surgery*.

[9] Alexander F (2007). Complications of ileal pouch anal anastomosis. *Seminars in Pediatric Surgery*.

[10] Tulchinsky H, Cohen CRG, Nicholls RJ (2003). Salvage surgery after restorative proctocolectomy. *British Journal of Surgery*.

[11] Körsgen S, Keighley MRB (1997). Causes of failure and life expectancy of the ileoanal pouch. *International Journal of Colorectal Disease*.

[12] Ogunbiyi OA, Korsgen S, Keighley MRB (1997). Pouch salvage: long-term outcome. *Diseases of the Colon and Rectum*.

[13] Saltzberg SS, DiEdwardo C, Scott TE, LaMorte WW, Stucchi AF, Becker JM (1999). Ileal pouch salvage following failed ileal pouch-anal anastomosis. *Journal of Gastrointestinal Surgery*.

[14] Zmora O, Efron JE, Nogueras JJ, Weiss EG, Wexner SD (2001). Reoperative abdominal and perineal surgery in ileoanal pouch patients. *Diseases of the Colon and Rectum*.

[15] Galandiuk S, Scott NA, Dozois RR (1990). Ileal pouch-anal anastomosis: reoperation for pouch-related complications. *Annals of Surgery*.

[16] Korsgen S, Nikiteas N, Ogunbiyi OA, Keighley MRB (1996). Results from pouch salvage. *British Journal of Surgery*.

[17] Herbst F, Sielezneff I, Nicholls RJ (1996). Salvage surgery for ileal pouch outlet obstruction. *British Journal of Surgery*.

[18] Delemarre JBVM (1994). Can (neo)rectal evacuation disorders be treated surgically in inflammatory bowel disease?. *Netherlands Journal of Medicine*.

[19] Read TE, Schoetz DJ, Marcello PW (1997). Afferent limb obstruction complicating ileal pouch-anal anastomosis. *Diseases of the Colon and Rectum*.

[20] Williams NS, Giordano P, Dvorkin LS, Huang A, Scott SM (2004). Full-thickness pouch prolapse after restorative proctocolectomy: a potential future problem treated by the new technique of external pelvic neorectal suspension (the Express procedure). *Diseases of the Colon and Rectum*.

[21] Ehsan M, Isler JT, Kimmins MH, Billingham RP (2004). Prevalence and management of prolapse of the ileoanal pouch. *Diseases of the Colon and Rectum*.

[22] Ng KH, Lim YK, Ho KS, Ooi BS, Eu KW (2009). Robotic-assisted surgery for low rectal dissection: from better views to better outcome. *Singapore Medical Journal*.

[23] Stefanidis D, Wang F, Korndorffer JR, Dunne JB, Scott DJ (2010). Robotic assistance improves intracorporeal suturing performance and safety in the operating room while decreasing operator workload. *Surgical Endoscopy*.

[24] Palep JH (2009). Robotic assisted minimally invasive surgery. *Journal of Minimal Access Surgery*.

